# Improving early intervention: identifying risk factors for UK military veterans that access military charities—a case-control study and an AI-powered predictive model

**DOI:** 10.1093/eurpub/ckaf140

**Published:** 2025-08-13

**Authors:** Giuseppe Serra, Federico Turoldo, Marco Tomietto, Andrew McGill, Matthew D Kiernan

**Affiliations:** Department of Nursing, Midwifery and Health, Faculty of Health and Life Sciences, Northumbria University, Newcastle upon Tyne, United Kingdom; Department of Medicine (DMED), University of Udine, Udine, Italy; Department of Medicine, Surgery and Health Sciences, University of Trieste, Trieste, Italy; Department of Nursing, Midwifery and Health, Faculty of Health and Life Sciences, Northumbria University, Newcastle upon Tyne, United Kingdom; Department of Nursing, Midwifery and Health, Faculty of Health and Life Sciences, Northumbria University, Newcastle upon Tyne, United Kingdom; Department of Nursing, Midwifery and Health, Faculty of Health and Life Sciences, Northumbria University, Newcastle upon Tyne, United Kingdom

## Abstract

Some veterans face unique physical, mental, and social challenges, leading them to seek assistance from military charities. This case-control study uses data from the MONARCH Study and the tri-service food insecurity study, with the aim to identify key risk factors associated with charity usage among UK veterans. Cases (veterans who accessed charities in 2022) were compared to controls (veterans who did not access charities). Logistic regression and a random forest algorithm were used to identify risk factors for charity use. Several risk factors for charity use were identified: younger age, living alone, being a non-officer, and living in rented accommodation. Having dependents was found to be protective but emerged as a risk factor for veterans living alone and protective for veterans living with others. The use of a random forest algorithm confirmed the statistical importance of these variables, offering deeper insights into complex interactions. These results improve our understanding of the risk factors for charity usage by veterans and provide a predictive model that could be implemented in planning service provision in public health. Additionally, it could be used as the basis for the implementation of targeted preventive interventions, allowing for proactive measures to be taken to support veterans before they reach a point of needing charity services in a period of crisis. These predictive models could enable more efficient resource allocation and the development of tailored strategies to address the specific needs of at-risk veteran subgroups.

## Introduction

### Complexity of the veteran population

Veterans are recognized as a population that can face increased challenges, and there is evidence that in recent years this vulnerability may be increasing [1]. While historically veterans had a longer life expectancy? than the general population, a phenomenon known as healthy soldier effect (HSE), in recent years, this advantage is eroding, possibly indicating a worsening of overall health status [1, 2].

In fact, some veterans may suffer the physical health consequences of risks related to military service, such as the chemical risk from exposure to explosives or gases [3], noise-induced hearing loss (explosions, heavy machinery), or biological risk derived from deployment in areas with a high prevalence of infectious disease. Moreover, the stress of being in combat leads some veterans to develop mental health conditions such as depression or Post-Traumatic Stress Disorder (PTSD) [4]. It has been argued that the worsening of health status may be associated with improved survival rates to injuries that in previous wars would have been un-survivable [5] (this may have caused an increase in veterans living with disabilities), or to the higher prevalence in younger veterans of risk factors such as smoking, drinking, and obesity [6, 7]. Beyond physical and mental health issues, veterans can face social challenges, including unemployment and financial difficulties [8].

Since the vulnerabilities of this population are multiple and affect different aspects of health, the needs of these individuals have been defined as complex [9]. An attempt to measure complexity in veterans has been made by Fadeeva *et al.*, who developed an indicator and validated it using military charity data [10, 11]. The results showed that, while the majority of veterans presented a low level of complexity, a significant minority had high levels.

### Assistance to veterans provided by charities

In the UK, veterans’ healthcare is provided by the NHS along with the rest of the general population. This is supplemented with over 1800 military charities that provide additional support for physical, mental, and social health issues [12]. Although military charity data are highly fragmented, the Map of Needs project [8] demonstrated significant informative potential. This initiative was further developed through the MONARCH Study [13], which ultimately established a comprehensive national registry of charity usage data from veterans in the UK.

The first analysis of the dataset unveiled the characteristics of veterans accessing charities, indicating they differ from the general veteran population as reported in the 2021 census. Specifically, veterans using charities tend to be younger, non-officers, living in rented accommodation, and often living alone [13]. While these findings provide valuable insights they are limited to charity users, so the conclusions remain purely hypothetical.

### The benefits of early intervention

Research indicates that veterans often refrain from seeking assistance until their problems become severe and unmanageable [14]. Therefore, it can be hypothesized that veterans that refer to a charity have struggled for a certain amount of time and with a certain number of issues before seeking help. As issues that affect veterans tend to influence and worsen one another [13], it is plausible that a single issue can escalate into a series of complex problems, making resolution significantly more challenging.

Consequently, early intervention for veterans is more beneficial and may be more cost-effective than allocating resources to assist veterans in crisis. It is possible that a simple intervention made at an early stage on healthy veterans could prevent a situation where a veteran is forced to seek help from a charity for complex needs.

However, implementing large-scale intervention measures is currently impractical for several reasons. First, there are approximately 2 million veterans in the UK [15, 16], necessitating an intervention of substantial scale. Second, early findings from the MONARCH Study demonstrate that only a small percentage of veterans [13] experience issues that lead them to seek help from military charities. Third, there is no validated intervention proven to prevent the specific physical, mental, and social health issues faced by veterans.

In addition, in recent years, the concept of Precision Public Health has emerged as a response to the increasing availability of extensive data, aiming to tailor public health interventions similarly to advancements in precision medicine. This approach seeks to enhance the effectiveness of public health strategies by using population-specific data to deliver targeted interventions at the right time and place [17–19].

### Previous evidence on risk factors for adverse outcomes

An alternative method of implementing an intervention would be to identify a subgroup with higher risk, to focus on veterans that could benefit the most. However, the risk factors associated with military charity usage remain unexplored, making it challenging to pinpoint a target population.

Johnson *et al.* [20] attempted to identify risk factors for adverse outcomes through a cross-sectional study assessing the prevalence of food insecurity among UK veterans. Their findings indicated that younger, non-officer veterans residing in rented accommodations were at higher risk compared to older, officer veterans owning their own homes. Nonetheless, the cross-sectional nature of the study did not allow for the determination of a definitive causal relationship.

### Objective of the study

The objective of this study is to determine the characteristics of military veterans which are risk factors for military charity usage, by comparing a group of veterans that accessed a charity (cases) and a group of veterans that did not (controls).

## Methods

### Study design

Case–control study.

### Setting and participants

Cases are selected by simple random sampling [21] among veterans who received services from at least one of the military charities included in the MONARCH dataset [13] between 1st of January 2022 and 31st of December 2022. The year 2022 was chosen because it was the most recent year with complete data from all charities included in the database. The selection was performed using the ‘sample’ function of basic R [22] (version 4.3.1), without replacement and using the seed 1234 to ensure reproducibility.

Controls are the veterans who were interviewed during the tri-service food insecurity study [20], a nationwide survey for which invitations were sent to all members of multiple veteran associations. Controls were selected only among those that reported never accessing a military charity for assistance.

The dataset of cases (15 007 records of benefits) was much larger than the dataset of controls (881 records). For this reason, only a sample of cases was used to maintain a 1:1 ratio between cases and controls. A flow diagram of cases and controls sampling and inclusion in the study is provided in Supplementary Fig. S1.

### Variables and data sources

The outcome of interest is a military veteran receiving at least one service from one of the military charities included in the MONARCH dataset in the study period. The source data come directly from the charities, where a means tested assessment accurately records each service provided for financial reporting purposes.

The exposures of interest are the socio-demographic characteristics and the service history of the veterans. For cases, this information is recorded by the charities before they provide the service. For controls, the information is gathered through the questionnaire used for the tri-service food insecurity study [20].

### Quantitative variables and groupings

The variables are defined as follows:

- Age. For cases, it is the difference in days between 1st of January 2022 and the date of birth, divided by 365.25 and rounded down. For controls, it is the rounded down age, in years, declared during of the data collection period (01 February 2023–31 March 2023) Age is categorized into 10-year intervals and further divided into working age (<66 years) and non-working age (≥66 years).
- Gender, rank, marital status, accommodation, and number of dependents are recorded by the charities for cases, while for controls, this information is gathered through a questionnaire.
- Only ‘male’ and ‘female’ genders were considered, with other declared genders excluded prior to sampling due to differing definitions in the two datasets.
- Rank was coded according to standard NATO definitions and categorized as ‘officer’ or ‘non-officer’.
- Marital status was classified into ‘married’, ‘cohabiting’, ‘in a civil relationship’, ‘single’, ‘divorced’, and ‘widowed’. The categories ‘married’, ‘cohabiting’, and ‘in a civil relationship’ were combined and used as a proxy for living with someone, while ‘single’, ‘divorced’, and ‘widowed’ were grouped and used as a proxy for living alone.
- Accommodation was categorized as ‘owner’ or ‘rented’, irrespective of the accommodation type (house, apartment, etc.).
- Dependents were classified based on the number of declared dependents. The variable was further grouped into ‘with dependents’ and ‘without dependents’

### Statistical analysis

Data were analysed using R software 4.3.1 [22].

Descriptive statistics were used to describe demographic data, socio-economic status, and other characteristics using the ‘gtsummary’ and ‘tableone’ package [23]. Categorical variables were presented as frequency, and continuous variables were presented as mean and standard deviation. Default tests were the Wilcoxon rank sum test for continuous variables, Pearson’s Chi-squared test without Yates’ correction for categorical variables with all expected cell counts ≥ 5, and Fisher’s exact test for categorical variables with any expected cell count < 5. Missing data were handled by listwise deletion. The statistical significance was set to < 0.05. Standardized mean difference (SMD) was also used to assess differences between subgroups, given the high numerosity of the sample. A value of SMD > 0.1 was considered significant.

Univariate and multivariate logistic regression were used to calculate odds ratios and 95% confidence intervals.

The multivariate model was built with variables that were significant in the univariate models, and consistent with similar models in available literature [20]. The variable ‘accommodation’ was removed due to the high number of missing values.

As part of the sensitivity analysis, five additional random samples were generated. Then, the models were compared to the results of the initial extraction to assess consistency.

As a secondary analysis, a random forest classification algorithm was fitted to the dataset to extrapolate the variable importance of each independent variable, and then compare the result with the multivariate logistic regression and explore any differences. The study dataset was used as a training set, while the test set consisted of 6864 complete cases from the MONARCH dataset that were not used in the training process.

The direction of the association was evaluated by comparing the predicted probability of being a case in different sub-categories. The strength of the association was evaluated using the Boruta algorithm [24], and the estimates of variable importance were expressed as mean decrease in accuracy. Multiple R packages were used for this secondary analysis [25–28].

To further explore the possible relationship between the number of dependents and living condition, a post-hoc analysis was performed, calculating a stratified logistic regression model by living condition.

### Ethical considerations

Data management was conducted in compliance with the General Data Protection Regulation (GDPR) (2018) and the UK Data Protection Act (2018). Ethical approval was obtained for both data sources [13, 20]. All personal information was removed from the datasets before making them available to the analysts.

## Results

Table 1 shows frequency distribution and characteristics of cases and controls.

**Table 1.** Frequency distribution and characteristics of cases and controls included in the study

Characteristic						
	Total	Controls	Cases	SMD^b^	SMD^b^	*P*-value^c^
	*N* = 1676^a^	*N* = 838^a^	*N* = 838^a^		*>0.1	
Gender				0.218	*	<0.001
Female	341 (20%)	134 (16%)	207 (25%)			
Male	1335 (80%)	704 (84%)	631 (75%)			
Age (grouped)				1.127	*	<0.001
18–29	42 (3%)	0 (0%)	42 (5%)			
30–39	148 (9%)	8 (1%)	140 (17%)			
40–49	155 (9%)	38 (5%)	117 (14%)			
50–59	270 (16%)	143 (17%)	127 (15%)			
60–69	417 (25%)	300 (37%)	117 (14%)			
70–79	346 (21%)	246 (30%)	100 (12%)			
≥ 80	281 (17%)	86 (10%)	195 (23%)			
Age (binary)				0.376	*	<0.001
< 66	842 (51%)	340 (41%)	502 (60%)			
≥ 66	817 (49%)	481(59%)	336 (40%)			
Living condition				0.920	*	<0.001
Living with others	881 (57%)	628 (76%)	253 (35%)			
Living alone	676 (43 %)	197 (24%)	479 (65%)			
Dependents				0.129	*	<0.001
0	310 (22%)	155 (19%)	155 (25%)			
≥ 1	1126 (78 %)	650 (81%)	476 (75%)			
Accommodation				0.777	*	<0.001
Owner	721 (80%)	678 (84%)	43 (49%)			
Rented	177 (20%)	133 (16%)	44 (51%)			
Service				0.637	*	<0.001
Army	890 (56%)	419 (50%)	471 (61%)			
Royal Air Force	300 (19%)	138 (16%)	162 (21%)			
Royal Marines	36 (2%)	0 (0%)	36 (5%)			
Royal Navy	372 (23%)	281 (34%)	91 (12%)			
Rank				0.626	*	<0.001
Non-officer	1244 (87%)	603 (78%)	641 (98%)			
Officer	186 (13%)	170 (22%)	16 (2%)			

a *n* (%); missing values are not shown.

b SMD: standardized mean difference.

c Pearson’s Chi-squared test; Fisher’s exact test.

Of the 838 cases (veterans who accessed a charity in 2022), 75% were male and 60% were working age. The most represented age groups were >80 (23%), 30–39 (17%), and 50–59 (15%). Most veterans were living alone (65%). Seventy-five percent had dependents and 51% lived in rented accommodation. The most represented service was Army (61%) followed by Royal Air Force (21%) and Royal Navy (12%). Ninety-eight percent 98% of cases were non-officers.

Of the 838 controls (veterans who declared not accessing a charity in 2022), 84% were male and 41% were in working age. The most represented age groups were 50–59 (17%), 60–69 (37%), and 70–79 (30%). Most veterans were living with others (76%), 81% had dependents and 85% were homeowners. The most represented service was Army (50%) followed by Royal Navy (34%) and Royal Air Force (16%). Seventy-eight percent of controls were non-officers.

All differences assessed were statistically significant.

Table 2 shows the results of univariate logistic regression predicting being a case. Results show that female gender was a risk factor for charity usage (OR 1.72; 95% CI 1.35–2.20), together with working age (OR 2.11; 95% CI 1.74–2.57), living alone (OR 6.03; 95% CI 4.85–7.54), living in rented accommodation (OR 5.22; 95% CI 3.29–8.28), and not being an officer (OR 11.29; 95% CI 6.90–19.83). Having at least one dependent was protective (OR 0.73; 95% CI 0.57–0.94), while the service was not significantly associated with being a case or a control.

**Table 2.** Univariate logistic regression predicting being a case

Characteristic	OR	(95% CI)
Gender		
M	Reference	
F	1.72	(1.35–2.20)
Age (binary)		
≥ 66	Reference	
< 66	2.11	(1.74–2.57)
Living condition		
Living with others	Reference	
Living alone	6.03	(4.85–7.54)
Accommodation		
Owner	Reference	
Rented	5.22	(3.29–8.28)
Rank		
Officer	Reference	
Non-officer	11.29	(6.90–19.83)
Service		
Army	Reference	
Royal Air Force	1.04	(0.80–1.28)
Royal Marines	>999	(0.11 – >999)
Royal Navy	0.28	(0.22–0.38)
Dependents		
0	Reference	
≥ 1	0.73	(0.57–0.94)

Table 3 shows the results of multivariate logistic regression predicting being a case. Results show that female gender was a risk factor for charity usage, but with more uncertain estimates than in the univariate models (OR 1.44; 95% CI 0.99–2.09). Being in working age (OR 2.99; 95% CI 2.21–4.07), a non-officer (OR 17.53; 95% CI 7.63–51.0) and living alone (OR 11.95; 95% CI 8.25–17.6) stayed associated with the risk of accessing a charity. Having dependents, which was a protective factor in the univariate analysis, became a risk factor (OR 3.73; 95% CI 2.44–5.77).

**Table 3.** Multivariate logistic regression and random forest algorithm predicting being a case

	Multivariate logistic regression		Random forest^a^	
Characteristic	OR	(95% CI)	Averaged predicted probability of being a case^b^	Variable importance^c^
Gender				4.17
M	Reference		0.26	
F	1.44	(0.99–2.09)	0.33	
Age (binary)				11.90
≥ 66	Reference		0.19	
< 66	2.99	(2.21–4.07)	0.35	
Rank				13.86
Officer	Reference		0.11	
Non-officer	17.53	(7.63–51.0)	0.32	
Living condition				30.22
Living with others	Reference		0.13	
Living alone	11.95	(8.25–17.6)	0.70	
Dependents				18.35
0	Reference		0.46	
≥ 1	3.73	(2.44–5.77)	0.34	

a Number of trees = 100. Out-of-bag estimate of error rate: 18.4%. Seed = 1234.

b Averaged predicted probabilities of being a case, as resulting from building a partial dependencies plot for the specified predictor variable.

c Mean decrease in accuracy was used as variable importance metrics. The higher the value, the higher the variable importance in the model.

A random forest model fitted to the final dataset and showed that all variables exhibited significant feature importance in predicting the probability of accessing a charity, as confirmed by the Boruta algorithm (Supplementary Fig. S2). Living condition was identified as the most important predictor (mean decrease in accuracy 30.22). To explore the direction of these associations, partial dependence plots (PDPs) were generated for each predictor, revealing the average predicted probabilities of being classified as a case if all data points assume that predictor value [26]. Results are shown as average predicted probability in Table 3. All variables were found to increase the probability of accessing a charity, accordingly to the multivariate logistic regression, since the predicted probability of being a case of the reference category was lower than the predicted probability of being a case of the category studied. On the contrary, having ≥1 dependents decreased the probability (0.34 vs 0.46) and can therefore to be considered a protective factor. The accuracy on the training set was 81.8%, the out-of-bag estimate of error rate was 18.4%, and the accuracy on the test set was 65.8%.

In the post-hoc analysis, logistic regression revealed the role of living condition as a qualitative multiplicative effect measure modifier (Table 4). Specifically, for veterans living with others, having dependents did not correlate with an increased probability of accessing charity services. However, for veterans living alone, the number of dependents emerged as a significant and strong predictor of charity access.

**Table 4.** Multivariate logistic regression predicting being a case by living condition

	Multivariate logistic regression		Multivariate logistic regression	
	Among veterans living with others		Living veterans living alone	
Characteristic	OR	(95% CI)	OR	(95% CI)
Gender				
M	Reference		Reference	
F	1.46	(0.82–2.60)	1.41	(0.80–2.47)
Age (binary)				
≥ 66	Reference		Reference	
< 66	2.02	(1.34–3.06)	5.03	(3.00–8.43)
Rank				
Officer	Reference		Reference	
Non-officer	20.6	(4.96–85.2)	17.7	(4.54–69.1)
Dependents				
0	Reference		Reference	
≥ 1	0.09	(0.04–0.25)	9.20	(5.51–15.4)

As for the sensitivity analysis, the tables created using different random samples produced results that were comparable to those obtained with the original one. The full results, including all variations and similarities, are presented in Supplementary Tables S1 and S2.

## Discussion

The findings reveal that younger, non-officer veterans living alone are at a substantially higher risk of accessing charity services compared to their older, officer counterparts living with others. This observation aligns with existing literature [13, 20].

The use of multivariate logistic regression allowed for the isolation of the individual contributions of various risk factors to the overall likelihood of charity usage. All factors remained significant except for gender, whose role remains ambiguous. Interestingly, having dependents was found to have a protective effect. Further stratification of the population by living condition revealed that this risk factor is only significant among veterans living alone.

Living condition acted as a confounder in the univariate analysis, as it was associated both with the predictor and the outcome. In fact, living with others is associated both with having dependents (i.e. children living at home) and with having less risk of being in crisis, as shown by previous studies [13]. This suggests that living condition plays a crucial role in how dependents influence the need for charity support among veterans. While veterans living with others may have additional support systems that mitigate the need for charity, veterans living alone with dependents face greater challenges, making them more likely to seek charitable assistance.

Additionally, the random forest algorithm confirmed these results and demonstrates that in this context it was a robust alternative to logistic regression. However, while the direction of the association is the same as in logistic regression, the rank of variable importance is not correspondent to the rank of the odds ratios. This can be explained by the fact that both models have a certain level of uncertainty, so the results should be evaluated jointly.

These results confirm once more the necessity of critical thinking in epidemiology and public health research. While statistics provide a framework for understanding relationships between variables, it is critical that these findings are interpreted with care. Without critical reasoning, there is a significant risk of oversimplifying complex interactions or misattributing causality. For example, in this study, the stratification by living condition allowed for the identification of the protective effect of dependents, which is particularly pronounced among veterans living with others, while for veterans living alone, it is a strong risk factor. This reinforces the notion that a one-size-fits-all approach in public health can be misleading.

The use of accurate and targeted data to improve public health, as opposed to a generalized approach, has been defined as ‘Precision Public Health’, aiming to provide ‘the right intervention to the right population at the right time’. This approach is considered by some to be a modernization of traditional epidemiology, leveraging advanced technologies and data analysis to achieve its goals [17–19]. In this study, the use of both logistic regression and random forest algorithms, together with the targeted subgroup analysis, constitutes a practical implementation of precision public health, showing how veteran charity usage data can lead ultimately to precisely targeted interventions.

### Strengths and limitations

One of the primary strengths of this study is the use of real-world veteran data from the MONARCH Study, which provides the most comprehensive representation available of the veteran population accessing charities in the UK.

Additionally, this study employs both classic epidemiologic measures and innovative machine learning algorithms. One of the key advantages of the random forest algorithm is its reduced sensitivity to collinearity among predictor variables, which can be a significant issue in logistic regression. This combined methodology allows the cross-validation of the results, leading to a more comprehensive understanding of phenomena than relying on a single method alone.

Another significant strength of this study is the development of a predictive model that, with further testing, could serve as a practical tool for forecasting charity usage.

However, there are several limitations to consider. The quality of the data used in the study may be variable, since cases and controls were collected in two different settings, and this could affect the accuracy of the findings. In addition, inconsistent or incomplete data entries could introduce bias or errors into the analysis.

The method of control selection—via questionnaire distributed to association members—may introduce selection bias, as this group may not be fully representative of the broader veteran population. However, key demographic indicators suggest reasonable representativeness: 59% of participants were over 66 years old, closely aligning with census data indicating 53% in that age group; similarly, the gender distribution in our sample (84% male) is comparable to census figures (86.4%) [29].

Similarly, while simple random sampling ensures our sample reflects the MONARCH dataset, it is possible that the MONARCH dataset, despite its large size, may not fully represent all UK charity users. However, this does not compromise the validity of our findings, as MONARCH remains the largest and most comprehensive dataset on charity users currently available.

Therefore, it may be assumed that the differences observed between cases and controls in Table 1 are less likely due to selection bias and more plausibly reflect true disparities in access to charitable services. Another limitation is the lack of prospective longitudinal testing. While the models demonstrate good accuracy on the training and test datasets, their performance has not yet been validated with healthy veterans. This limits the generalizability of the findings. Additionally, although the study attempts to control for various confounders, there may still be unmeasured variables that could influence the results. Factors such as mental health status or social support networks were not included in the analysis but could significantly impact charity service usage [30].

The interpretability of machine learning models also poses a challenge. While the random forest algorithm provides robust predictive power, the importance of individual variables in the model is less interpretable compared to logistic regression. This makes it difficult to draw specific conclusions about the role of each predictor. The problem was addressed in this study by comparing the two algorithms, which provided a more detailed picture of the contribution of each risk factor.

## Conclusions

This study makes a significant contribution to understanding the risk factors that lead UK veterans to use charity services, by integrating both traditional logistic regression and an innovative machine learning approach to analyse the data.

Future research should continue to explore the roles of these variables to better inform intervention programs. The predictive models developed in this study hold significant potential for real-world application, enabling the estimation of the likelihood that a healthy veteran will access charity services within a year. This capability is the basis for the implementation of targeted preventive interventions, allowing for proactive measures to be taken to support veterans before they reach a point of needing charity services in a period of crisis. By leveraging these models, policymakers and service providers can allocate resources more efficiently and develop tailored strategies to address the specific needs of at-risk veteran subgroups.

## Supplementary Material

ckaf140_Supplementary_Data

## Data Availability

The dataset used for this study is available upon request from the corresponding author, subject to approval of original studies’ principal investigator [13, 20]. Key points Younger, non-officer, single veterans are more likely to access military charities. Predictive models potentially can identify veterans at higher risk of charity use. Targeted, data-driven interventions could efficiently allocate resources to specific subgroups of veterans and prevent them from needing charity services.
