# Supplementary material for: Improving early intervention: identifying risk factors for UK military veterans that access military charities—a case-control study and an AI-powered predictive model
Source: Eur J Public Health. 2025 Aug 13;35(5):867–72. doi: 10.1093/eurpub/ckaf140 (PMC12529282; doi:10.1093/eurpub/ckaf140)
Supplement: ckaf140_Supplementary_Data [file ckaf140_supplementary_data.docx]

# Supplementary Material

# Improving early intervention: Identifying risk factors for UK military veterans that access military charities– a case-control study and an AI-powered predictive model

Giuseppe Serra^1,2^, Federico Turoldo^3^, Marco Tomietto^1,*^, Andrew McGill^1^, Matthew D Kiernan^1^

1. *Northumbria University, Department of Nursery, Midwifery and Health, Newcastle upon Tyne, United Kingdom*
2. *University of Udine, Department of Medicine (DMED), Udine, Italy*
3. *University of Trieste, Department of Medicine, Surgery and Health Sciences, Trieste, Italy*

*Corresponding author

Marco Tomietto, Department Nursing, Midwifery and Health, Faculty of Health and Life Sciences, Northumbria University, Newcastle upon Tyne, United Kingdom.

e-mail: [marco.tomietto@northumbria.ac.uk](mailto:marco.tomietto@northumbria.ac.uk); telephone number: +44 (0191) 215 6060

**Figure S1. Flow diagram of cases and controls sampling and inclusion in the study.**

**CONTROLS**

**CASES**

881 unique responders to the questionnaire
FOOD INSECURITY

15,007 subjects in MONARCH dataset helped by charities in 2022

28 subjects declared receiving befits by a charity; 853 subjects remaining

Variable harmonizing

13 subjects excluded due to missing gender; 14,994 subjects remaining

2 subjects did not answer the question about benefits received by charities; 851 subjects remaining

13 subjects excluded due to missing gender; 838 subjects remaining

Sampling: 838 subjects
seed(1234)

RECORDS: 1676

838 cases

838 controls

**Figure S2: Boruta feature Importance**


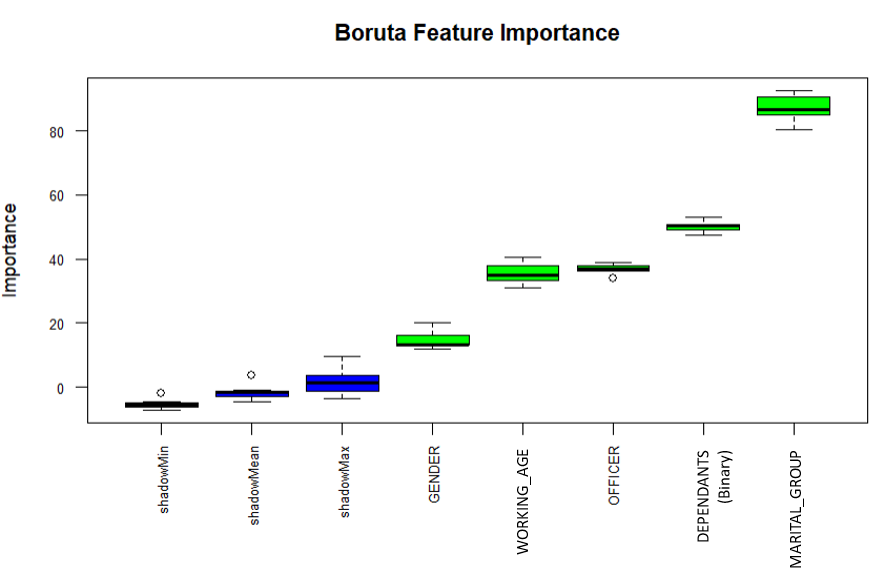


**Table S1: Sensitivity analysis: Frequency distribution and characteristics of cases sampled with 5 different random seeds.**

| **Characteristic** | **CASES**  N=838*^1^* | **SMD**^2^ | **CASES**  N=838*^1^* | **SMD**^2^ | **CASES**  N=838*^1^* | **SMD**^2^ | **CASES**  N=838*^1^* | **SMD**^2^ | **CASES**  N=838*^1^* | **SMD**^2^ |
| --- | --- | --- | --- | --- | --- | --- | --- | --- | --- | --- |
| **RANDOM SEED** | **(99999)** | | **(88888)** | | **(77777)** | | **(66666)** | | **(55555)** | |
| **GENDER** |  | 0.226 |  | 0.264 |  | 0.193 |  | 0.267 |  | 0.223 |
| FEMALE | 210 (25%) |  | 224 (27%) |  | 198 (24%) |  | 225 (27%) |  | 209 (25%) |  |
| MALE | 628 (75%) |  | 614 (73%) |  | 640 (76%) |  | 613 (73%) |  | 629 (75%) |  |
| **AGE GROUP** |  | 1.103 |  | 1.170 |  | 1.189 |  | 1.213 |  | 1.174 |
| 10-29 | 38 (5%) |  | 30 (4%) |  | 51 (6%) |  | 44 (5%) |  | 34 (4%) |  |
| 30-39 | 130 (15%) |  | 149 (18%) |  | 137 (16%) |  | 137 (16%) |  | 140 (17%) |  |
| 40-49 | 130 (15%) |  | 122 (15%) |  | 135 (16%) |  | 116 (14%) |  | 126 (15%) |  |
| 50-59 | 108 (13%) |  | 126 (15%) |  | 105 (13%) |  | 131 (16%) |  | 141 (17%) |  |
| 60-69 | 111 (13%) |  | 103 (12%) |  | 109 (13%) |  | 104 (12%) |  | 100 (12%) |  |
| 70-79 | 121 (14%) |  | 98 (12%) |  | 101 (12%) |  | 87 (10%) |  | 97 (12%) |  |
| ≥ 80 | 200 (24%) |  | 210 (22%) |  | 200 (24%) |  | 219 (26%) |  | 200 (24%) |  |
| **WORKING AGE** |  | 0.314 |  | 0.349 |  | 0.369 |  | 0.341 |  | 0.386 |
| < 66 | 477 (57%) |  | 491 (59%) |  | 499 (59%) |  | 488 (58%) |  | 506 (60%) |  |
| ≥ 66 | 361 (43%) |  | 347 (41%) |  | 339 (41%) |  | 350 (42%) |  | 332 (40%) |  |
| **LIVING CONDITION** |  | 0.891 |  | 0.920 |  | 0.927 |  | 0.893 |  | 0.852 |
| Living with others | 267 (36%) |  | 250 (35%) |  | 249 (34%) |  | 255 (36%) |  | 267 (37%) |  |
| Living alone | 481 (64%) |  | 473 (65%) |  | 477 (66%) |  | 461 (64%) |  | 449 (63%) |  |
| **DEPENDANTS** |  | 0.084 |  | 0.076 |  | 0.108 |  | 0.176 |  | 0.146 |
| 0 | 140 (23%) |  | 141 (23%) |  | 149 (24%) |  | 159 (27%) |  | 160 (25%) |  |
| ≥ 1 | 478 (77%) |  | 490 (77%) |  | 480 (76%) |  | 438 (73%) |  | 472 (75%) |  |
| **RENTING** |  | 0.573 |  | 0.619 |  | 0.764 |  | 0.643 |  | 0.539 |
| Owner | 51 (59%) |  | 52 (57%) |  | 34 (50%) |  | 51 (55%) |  | 56 (60%) |  |
| Rented | 36 (41%) |  | 40 (43%) |  | 34 (50%) |  | 41 (45%) |  | 37 (40%) |  |
| **SERVICE** |  | 0.631 |  | 0.685 |  | 0.716 |  | 0.617 |  | 0.674 |
| Army | 480 (62%) |  | 507 (66%) |  | 486 (63%) |  | 455 (61%) |  | 509 (67%) |  |
| Royal Air Force | 164 (21%) |  | 139 (18%) |  | 152 (20%) |  | 159 (21%) |  | 124 (16%) |  |
| Royal Marines | 30 (4%) |  | 37 (5%) |  | 43 (6%) |  | 40 (5%) |  | 37 (5%) |  |
| Royal Navy | 88 (11%) |  | 78 (10%) |  | 73 (10%) |  | 91 (12%) |  | 80 (11%) |  |
| Other | 11 (1%) |  | 11 (1%) |  | 14 (2%) |  | 3 (0.4%) |  | 8 (1%) |  |
| **OFFICER** |  | 0.573 |  | 0.609 |  | 0.621 |  | 0.607 |  | 0.575 |
| Non-Officer | 619 (96%) |  | 625 (97%) |  | 613 (98%) |  | 648 (97%) |  | 626 (96%) |  |
| Officer | 23 (4%) |  | 18 (3%) |  | 16 (2%) |  | 19 (3%) |  | 23 (4%) |  |
| *^1^* n (%); Missing values are not shown.  ^2^ SMD: Standardized Mean Difference with reference to controls, whose characteristic are available in Table1. | | | | | | | | | | |

**Table S2. Sensitivity analysis: Multivariate logistic regression predicting being a case with 5 different random seeds used for sampling cases.**

| **Characteristic** | **Logistic regression**  OR  (95%CI) | **Logistic regression**  OR  (95%CI) | **Logistic regression**  OR  (95%CI) | **Logistic regression**  OR  (95%CI) | **Logistic regression**  OR  (95%CI) |
| --- | --- | --- | --- | --- | --- |
| **RANDOM SEED** | **(99999)** | **(88888)** | **(77777)** | **(66666)** | **(55555)** |
| **Gender** |  |  |  |  |  |
| M | Ref. | Ref. | Ref. | Ref. | Ref. |
| F | 1.33  (0.91-1.95) | 1.66  (1.14-2.42) | 1.16  (0.79-1.69) | 1.61  (1.12-2.33) | 1.36  (0.93-1.97) |
| **Working_Age** |  |  |  |  |  |
| ≥ 66 | Ref. | Ref. | Ref. | Ref. | Ref. |
| < 66 | 2.66  (1.96-3.60) | 3.18  (2.32-4.36) | 3.09  (2.27-4.19) | 3.11  (2.28-4.22) | 4.19  (3.06-5.75) |
| **Officer** |  |  |  |  |  |
| Officer | Ref. | Ref. | Ref. | Ref. | Ref. |
| Non-Officer | 7.49  (3.84-14.6) | 14.0  (5.88-33.3) | 22.0  (7.85-61.4) | 14.1  (6.00-33.2) | 15.3  (6.41-36.3) |
| **LIVING CONDITION** |  |  |  |  |  |
| Living with others | Ref. | Ref. | Ref. | Ref. | Ref. |
| Living alone | 13.8  (9.43-20.2) | 14.0  (9.47-20.7) | 11.0  (7.56-15.9) | 11.2  (7.72-16.2) | 11.0  (7.50-16.2) |
| **Dependants** |  |  |  |  |  |
| 0 | Ref. | Ref. | Ref. | Ref. | Ref. |
| ≥ 1 | 3.66  (2.39-5.62) | 3.79  (2.45-5.87) | 2.90  (1.90-4.43) | 2.80  (1.85-4.25) | 3.01  (1.96-4.63) |
